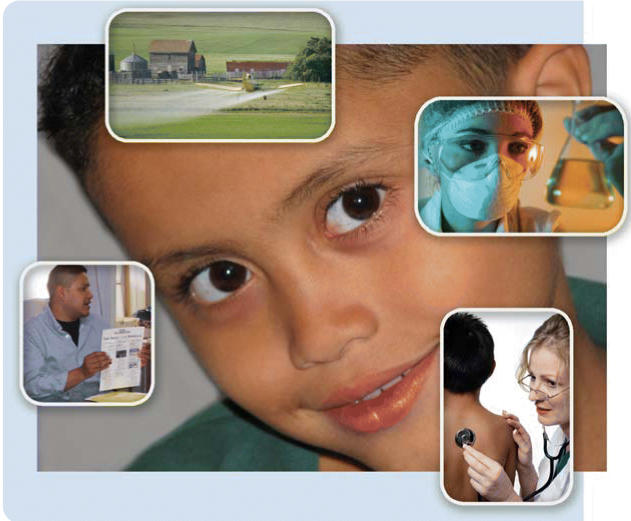# Community Outreach and Translation Core: Translating Research Findings to Improve the Health of Our Children

**Published:** 2006-06

A key goal of the Centers for Children’s Environmental Health and Disease Prevention Research is translating basic research findings into intervention and prevention methods to enhance awareness among communities, health care professionals, and policy makers of environmentally related diseases and health conditions. In 2003, research translation was formalized with the establishment of a Community Outreach and Translation Core (COTC) in each Center.

A COTC develops, implements, and evaluates strategies to translate and apply the Center’s scientific findings into information that can be used to protect the health of children. Members bring together diverse expertise to communicate research findings to the intended audiences. COTCs employ a variety of methodologies, from very basic communication strategies to intensive capacity-building exercises to achieve their goals. Following are examples of outreach strategies.

The University of Southern California COTC operates a program where researchers train residents to use air monitoring equipment. Such capacity building enables residents to participate in the research process.

The University of Washington COTC partners with a Pediatric Environmental Health Specialty Unit to conduct continuing education courses for health care professionals who work with children and parents. Center investigators present their research at these courses.

The Columbia Center applied its research findings to an Integrated Pest Management Intervention in New York City low-income public housing, convincing city officials to replace traditional extermination methods in public housing with lower toxic techniques.

COTCs inform families in their studies and throughout local neighborhoods of their research and other health findings through low-literate print materials, which also offer tips for reducing exposures to harmful environmental pollutants at home and in the community.

COTCs help advance the rate at which the Centers for Children’s Environmental Health translate their research to improve the health of children. Even still, there exist opportunities to improve, enhance, and coordinate COTC efforts to have an even greater impact at the local and national level.

For more information on these centers or to contact the program officials see : **http://www.niehs.nih.gov/translat/children/children.htm**

## Contacts

**Kimberly Gray, Ph.D.** | gray6@niehs.nih.gov

**Annette Kirshner, Ph.D.** | Kirshner@niehs.nih.gov

**Cindy Lawler, Ph.D.** | lawler@niehs.nih.gov

**Liam O’Fallon** | ofallon@niehs.nih.gov

## Figures and Tables

**Figure:**